# Sagittal and Vertical Craniofacial Growth Pattern and Timing of Circumpubertal Skeletal Maturation: A Multiple Regression Study

**DOI:** 10.1155/2016/1728712

**Published:** 2016-11-22

**Authors:** Giuseppe Perinetti, Luigi Rosso, Riccardo Riatti, Luca Contardo

**Affiliations:** Department of Medical, Surgical and Health Sciences, School of Dentistry, University of Trieste, Trieste, Italy

## Abstract

The knowledge of the associations between the timing of skeletal maturation and craniofacial growth is of primary importance when planning a functional treatment for most of the skeletal malocclusions. This cross-sectional study was thus aimed at evaluating whether sagittal and vertical craniofacial growth has an association with the timing of circumpubertal skeletal maturation. A total of 320 subjects (160 females and 160 males) were included in the study (mean age, 12.3 ± 1.7 years; range, 7.6–16.7 years). These subjects were equally distributed in the circumpubertal cervical vertebral maturation (CVM) stages 2 to 5. Each CVM stage group also had equal number of females and males. Multiple regression models were run for each CVM stage group to assess the significance of the association of cephalometric parameters (ANB, SN/MP, and NSBa angles) with age of attainment of the corresponding CVM stage (in months). Significant associations were seen only for stage 3, where the SN/MP angle was negatively associated with age (β coefficient, −0.7). These results show that hyperdivergent and hypodivergent subjects may have an anticipated and delayed attainment of the pubertal CVM stage 3, respectively. However, such association remains of little entity and it would become clinically relevant only in extreme cases.

## 1. Introduction

The knowledge of the associations between the timing of skeletal maturation and craniofacial growth is of primary importance when planning a functional treatment for most of the skeletal malocclusions, including those on the sagittal [1] and vertical dimensions [2, 3]. Although being a controversial issue [4–6], functional treatment for Class II malocclusion would induce clinically relevant mandibular elongation when performed during the pubertal growth phase [7, 8], while, Class III malocclusion requires early treatment [1]. Finally, both excessive vertical facial growth [2] and deepbite [3] have also been reported to be best treated during the pubertal growth phase. These aspects are of particular importance also in consideration that skeletal Class III malocclusion [9] and vertical facial growth pattern [10] tend to aggravate when not treated. Therefore, the knowledge of whether attainment of a specific growth phase is also dependent on the different sagittal and vertical craniofacial growth pattern has a clinical relevance in terms of timing of intervention. In this regard, the most common procedures to monitor the different growth phases are the radiographic methods of maturational stages of the cervical vertebral maturation (CVM) [1, 11] and hand-and-wrist maturation (HWM) (for review, see [12]).

To date very little research has focused on the possible association between the timing of the circumpubertal skeletal maturation phases and sagittal craniofacial growth, that is, skeletal class [13–15]. Moreover, none of these previous studies investigated possible associations of vertical craniofacial facial growth and timing of attainment of skeletal maturation phases. These studies were further limited by the use of univariate analyses [14, 15] with only one exception, where a multivariate model was used [13]. Finally, a further study [16] used an overall craniofacial composite measured, derived from multiple measurements; thus, it was not able to discriminate between sagittal and vertical growth patterns. Investigation on the craniofacial vertical growth pattern and timing of skeletal maturation becomes of interest also in consideration of the previous evidence reporting an earlier dental maturation in hyperdivergent subjects [17].

Therefore, through multivariate models, this cross-sectional study was aimed at evaluating whether sagittal and vertical craniofacial growth pattern, as described by common cephalometric parameters, has an association with the timing of circumpubertal skeletal maturation, that is, age of attainment of the maturation phases as defined by the CVM method.

## 2. Materials and Methods

### 2.1. Study Population and Design

The database between January 2009 and December 2015 of the Sections of Stomatology of the Department of Medical, Surgical and Health Sciences, University of Trieste, was screened. This study included subjects who were seeking orthodontic treatment and who had never been treated before. As a routine procedure, a signed informed consent for releasing diagnostic material for scientific purposes was obtained from the patients' parents prior to entry into treatment, procedures followed adhered to the World Medical Organization Declaration of Helsinki [18], and the protocol was reviewed and approved by the local Ethical Committee. In particular, in the first clinical session a lateral cephalograms was taken as a part of the pretreatment clinical recording. The following inclusion criteria were applied: (i) age between 7 and 17 years; (ii) circumpubertal skeletal maturation between CVM stages 2 and 5; (iii) absence of any craniofacial anomaly or extensive dental caries or restorations; (iv) good general health with no signs of symptoms of temporomandibular disorders; (v) no history of trauma at the craniofacial region; and (vi) Caucasian ethnicity. A dedicated X-ray machine (KODAK 8000C; Eastman Kodak Company) was employed for the recording of lateral head cephalograms. Settings were of 73–77 kV, 12 mA with an exposure time of 0.80 seconds. Images were saved at 300 dpi resolution and radiographs of low quality were excluded. An experienced orthodontist (LC) assisted by a second operator (LR) screened the cases for inclusion. A further experienced orthodontist (GP) was involved to ensure correct enrollment and, in case of disagreement, discussion was made until satisfaction of both operators. From an initial sample of over 450 subjects, total of 320 subjects (160 females and 160 males) were included in the study (mean age, 12.3 ± 1.7 years; range, 7.6–16.7 years).

### 2.2. Cephalometric Analysis for the Face and Cervical Vertebrae

**Figure 1:**
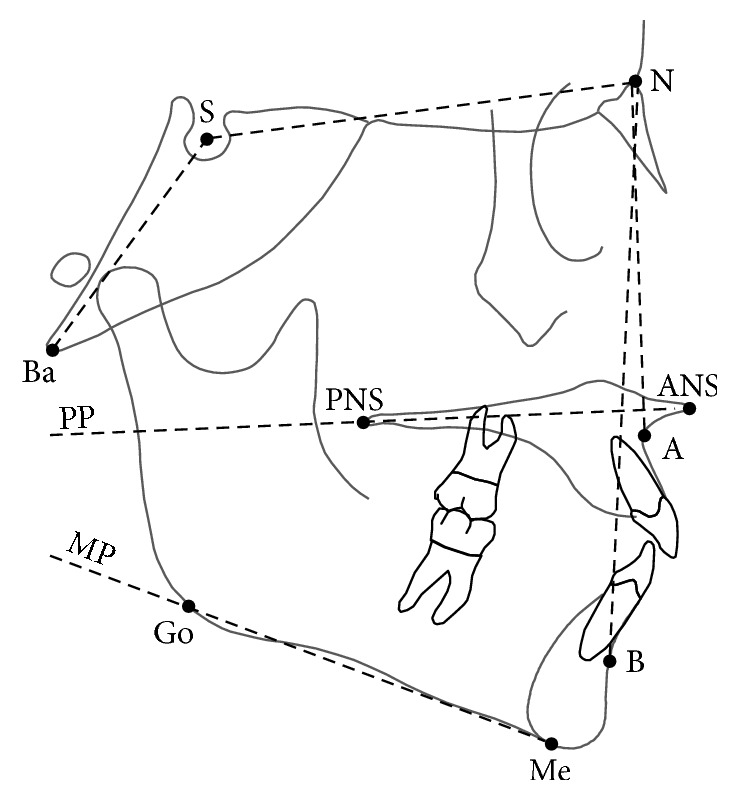
Diagram of the cephalometric measurements of the craniofacial complex. Landmarks: A, subspinale; B, supramentale; N, nasion; S, centre of the sella turcica; Ba, Basion; ANS, anterior nasal spine; PNS, posterior nasal spine; Me, menton; Go, Gonion; Planes: PP, palatal plane; MP, mandibular plane. See text for details.

A customized digitization regimen and analysis with cephalometric software (Viewbox, version 3.0, dHAL Software, Kifissia, Greece) were used for all cephalograms examined in this study. The cephalometric analysis of the face required the digitization of 9 landmarks (Figure 1) [19]. The customized cephalometric analysis included 4 angular measurements as follows (Figure 1): maxillary prognathism (SNA angle), mandibular prognathism (SNB angle), maxillomandibular relationship (ANB angle), maxillary inclination relative to the cranial base (SN/PP angle), mandibular inclination relative to the cranial base (SN/MP angle), and cranial base angle (NSBa angle).

**Figure 2:**
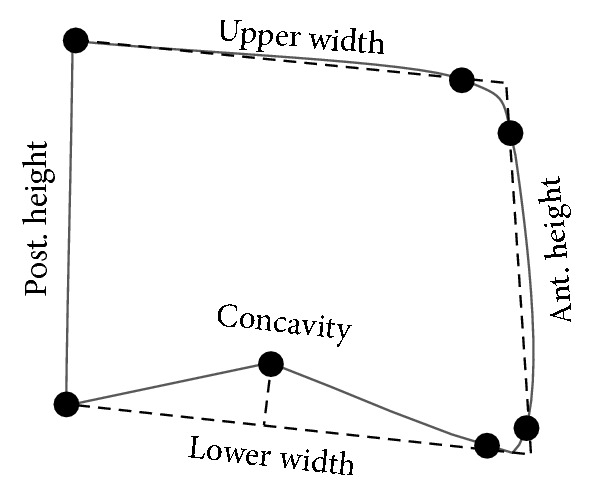
Diagram of the cephalometric measurements of the cervical vertebrae. Only a vertebral body is shown for clarity. In cervical vertebra 2, only concavity was measured. See text for details.

Regarding the body of the cervical vertebrae, a quantitative assessment of the shape (maturation) was also performed. The customized cephalometric analysis included measurements generated from 17 landmarks from which 11 linear and 8 angular variables were derived. Among the linear variables, 3 were for concavities of lower borders of C2–C4 and 8 related to the anterior and posterior heights and upper and lower widths for the C3 and C4 (Figure 2) [20]. Among angular variables, 4 were for the inner angles of the C3 and other 4 were for the inner angles of the C4. The data were used to calculate presence/absence of concavity and shape of the vertebral body through a dedicated Excel data sheet where linear and angular measurements were used in combination. The used method was independent of absolute recordings; instead we used relative dimensions to assess the shapes of the C3 and C4, while the concavity was assessed when it was at least 10% of the corresponding posterior height of the cervical body. The posterior height of the C3 was considered when assessing the concavity in the C2. Each CVM stage was retrieved according to the concavities of the C2–C4 and shapes of the C3 and C4 as reported below.

Lateral cephalograms were standardized as to real dimensions, that is, magnification factor of 0%. All of cephalograms were traced by a last year resident (LR), and a second investigator (GP) checked each tracing for accuracy. Both the Viewbox.vbr and Excel worksheet.xlsx files are available upon request to the corresponding author.

### 2.3. Cervical Vertebral Maturation Assessment

The CVM method according to Baccetti et al. [1] with minor modifications has been applied herein. The method has 6 stages: 2 prepubertal (1 and 2), 2 pubertal (3 and 4), and 2 postpubertal (5 and 6). These stages were briefly defined as follows:* stage 1*, when the lower borders of the second, third, and fourth vertebrae (C2, C3, and C4) are flat and the bodies of C3 and C4 are trapezoid in shape;* stage 2*, when only the lower border of C2 is concave and the bodies of C3 and C4 are trapezoid;* stage 3*, when the lower borders of C2 to C3 have concavities and the bodies of C3 and C4 are either trapezoid or rectangular horizontal in shape. Alternatively, when the concavity is present only at the lower border of the C3 with the bodies of C3 and C4 either trapezoid or rectangular horizontal in shape;* stage 4*, when the lower borders of C2 to C4 have concavities and the bodies of both C3 and C4 are both rectangular horizontal or at least one rectangular horizontal and the other trapezoidal;* stage 5*, when the lower borders of C2 to C4 have concavities, and at least one or both of the bodies of C3 and C4 are squared. Alternatively, when at least the body of either C3 or C4 is squared with a lack of concavity at the lower border in either C3 or C4;* stage 6*, when the lower borders of C2 to C4 have concavities, and at least one or both of C3 and C4 are rectangular vertical. Exceptional cases, that is, outside the reported norms, were managed as previously reported [20].

### 2.4. Method Error

With the aim of quantifying the full method error of the recordings for each recorded parameter, the method of moments variance estimator [21] was used on a random sample of 20 replicate measurements. Therefore, the mean error and 95% confidence intervals (CIs) between the repeated recordings were calculated using the MME variance estimator. Moreover, the repeatability in the CVM stage assignment in the same pairs of measurements was evaluated using the percentage of agreement and by both unweighted and linear weighted kappa coefficients presented as mean and 95% CI. The kappa coefficient ranges from zero for no agreement to 1 for perfect agreement [22].

### 2.5. Statistical Analysis

The SPSS software version 20 (SPSS® Inc., Chicago, IL, USA) and the G^*∗*^Power software version 3.1.9.2 (http://www.gpower.hhu.de/en.html) were used to perform the subsequent data analysis. After testing the normality of the data with the Shapiro-Wilk test and Q-Q normality plots of the residuals and the equality of variance among the datasets using a Levene test, parametric methods were used for data analysis [23]. The significance of the difference in each craniofacial and cervical vertebral cephalometric parameter among the CVM stage groups was evaluated through a one-way analysis of variance [23].

Moreover, within each CVM stage group, the association of each of the craniofacial parameters (explanatory variables) with the chronological age in months (dependent variable) was investigated by means of backward multiple linear regressions. In particular, a bivariate correlation matrix with Pearson coefficient was executed for each CVM stage group including all the craniofacial cephalometric parameters, according to which the SNB and SN/PP angles were excluded from the multivariate models. Thus, explanatory variables were sex (male), SNA angle, ANB angle, SN/MP angle, and NSBa angle. The cut-off levels of significance used were 0.01 and 0.05 for entry and removal, respectively. For each multiple regression model, multicolinearity among the remaining explanatory variables was also again checked for through the tolerance and variance inflation factor parameters. Finally, in* a posteriori* power analysis with 80 cases* per* model, considering an *F* ^2^ equal to 0.15, an alpha level of 0.05 and with 5 explanatory variables, the resulting power was 92.8%.

A *p* < 0.05 was used for rejection of the null hypothesis.

## 3. Results

For the face measurements, greatest method error of 1.06° (0.81–1.55) was for the SN/MP angle. For the cervical vertebrae measurements, greatest method errors were 0.18 mm (0.14–0.27), 0.24 mm (0.18–0.36), and 1.94° (1.48–2.84), for the concavities, linear, and angular measurements, respectively. The overall percentage of agreement for the CVM stages was 90% (18 cases out of 20). The unweighted kappa coefficient was 0.82 (0.71–1), and the weighted kappa coefficient was 0.93 (0.84–1).

Chronological ages for each group according to the sexes are reported in Table 1. For females, mean ages ranged from 10.1 to 12.7 years in CVM stage 2 and CVM stage 5 groups, respectively. For males, mean ages ranged from 11.4 to 14.1 years in CVM stage 2 and CVM stage 5 groups, respectively. The difference between the sexes within each group was significant (*p* = 0.000, each).

Descriptive statistics for each analysed parameter is reported in Table 2. The SNA angle ranged from 80.3° ± 3.2 (CVM stage 4) to 81.2° ± 3.4 (CVM stage 5); the SNB angle ranged from 76.7° ± 3.7 (CVM stage 4) to 77.7° ± 3.7 (CVM stage 5); the ANB angle ranged from 3.5° ± 2.3 (CVM stage 5) to 3.9° ± 2.1° (CVM stage 3); the SN/PP angle ranged from 7.1° ± 3.9 (CVM stage 3) to 8.1°  ± 2.8 (CVM stage 5); the SN/MP angle ranged from 30.4° ± 5.9 (CVM stage 3) to 31.2° ± 5.6 (CVM stage 2); the NSBa angle ranged from 129.4° ± 4.6 (CVM stage 3) to 130.7° ± 4.7 (CVM stage 5). For all of these craniofacial cephalometric parameters the differences among the groups were not statistically significant.

Results of the backward multiple linear regression models according to each CVM stage group are reported in Table 3. In the CVM stage 2 group (Model 1) *R* ^2^ was of 0.213 with the sex (male) and ANB angle positively associated with the age of attainment of the CVM stage 2 with *β* coefficients of 17.0 and 1.3, respectively. However, only the sex reached the statistical significance (*p* = 0.000), while the ANB angle did not (*p* = 0.077). In the CVM stage 3 group (Model 2) *R* ^2^ was of 0.269 with the sex (male) and SN/MP angle positively and negatively associated with *β* coefficients of 13.6 and −0.7, respectively (*p* = 0.015, at least). In the CVM stage 4 group (Model 3) *R* ^2^ was of 0.194 with the sex (male) and NSBa angle positively associated with the age of attainment of the CVM stage 4 with *β* coefficients of 15.4 and 0.6, respectively. However, only the sex reached the statistical significance (*p* = 0.000), while the NSBa angle did not (*p* = 0.093). Finally, In the CVM stage 5 group (Model 4) *R* ^2^ was of 0.165 with only the sex (male) positively associated with the age of attainment of the CVM stage 5 with a *β* coefficient of 16.3 (*p* = 0.000).

## 4. Discussion

Through multivariate models, the present study demonstrates a little association of the sagittal and vertical craniofacial growth pattern with the timing of skeletal maturation. While females had anticipated attainment of each CVM stage as compared to males (Table 1), the different cephalometric parameters showed no significant differences among the CVM stage groups (Table 2), allowing a more reliable comparison of the regression models.

The previous investigations [13–15] on sagittal craniofacial growth pattern and timing of skeletal maturation were focused on the CVM stages 3 and 4. Therefore, present data on the timing of the CVM stages 2 and 5 are not comparable with previous evidence. Of interest, *R* ^2^ retrieved for the models ranged from 0.165 to 0.269 (Table 3). Although such values were not particularly high, the greatest value was seen for the pubertal CVM stage 3 while, generally, the values decreased as maturation progresses into the postpubertal phases. Thus, in spite of the significant associations, the different CVM stages, sex, and craniofacial parameters all together accounted for no more than ≈27% of the total variability of corresponding ages. This evidence demonstrates how other relevant factors are responsible for the timing of skeletal maturation such as genetics, ethnicity, nutrition, and socioeconomic status [24].

As expected, sex was the most significant factor associated with the age of attainment of each CVM stage from 2 to 5 (Tables 1 and 3). According to the *β* coefficients, the male subjects had on average a delayed attainment of the different stages about 15 months later as compared to females. This evidence is in line with previous studies using the CVM [1] or other radiographic maturational methods [25, 26].

Herein, the ANB, SN/MP, and NSBa angles yielded the most relevant associations with the mean age for the attainment of the CVM stages 2, 3, and 4, respectively (Table 3). In particular, the greater the ANB angle, the greater the mean age for the attainment of the CVM stage 2, while, the greater the MP/SN angle, the lower the age for the attainment of the CVM stage 3; finally, the greater the NSBa angle, the greater the age for the attainment of the CVM stage 4. However, only the SN/MP angle yields an association that reached a statistically significant level (*p* = 0.015), while the ANB and NSBa angles yielded association very close to the significance level (*p* < 0.1), according to which they were kept in the final regression models. According to the *β* coefficients, unitary increments in ANB angle would account for about 1.6 months' retardation in the attainment of the CVM stage 2; unitary increments in SN/MP would account for about 0.7 months' anticipation of the attainment of the CVM stage 3, and unitary increments of the NSBa angle would account for about 0.6 months' anticipation of the attainment of the CVM stage 4. However, the relevance on the ANB angle in the age of attainment of the CVM stage 2 would also be limited by the concept that, from a clinical standpoint, the attainment of the pubertal CVM stages 3 and 4 is of primary importance in most of the functional treatments [1].

It has been suggested that the deficiency [27] and increased [9] mandibular length in Class II and Class III subjects at the pubertal growth spurt could be linked to the different duration of the pubertal peak in these subjects, as compared to those of Class I subjects [13–15]. Indeed, shorter and longer pubertal growth spurt, as recorded through the ages of attainment of CVM stages 3 and 4, have been reported for untreated Class II [15] and Class III [14] subjects, respectively.

The present results on the ANB angle and age of attainment of the CVM stage 2 group, although not statistically significant, are consistent with previous evidence showing that 8- to 14-year-old subjects with Class II malocclusion exhibited twice as much chance of being in CVM stage 1 or 2 than individuals with Class I malocclusion with similar age [13]. Regarding the pubertal stages, the duration of the maturation from CVM stage 3 to stage 4 has been reported for Class II subjects to be about 4 months shorter as compared to that of Class I subjects [15]. The present results do not support such evidence, with the CVM stages 3 and 4 not showing association with the craniofacial sagittal growth pattern. Differences in the study designs may explain such inconsistency (see also below).

In a previous investigation [14], the average age at onset of the pubertal peak was very similar for both skeletal Class I and Class III subjects. Therefore, the present data on the CVM stage 3 would be consistent with the concept that the sagittal growth has no influence on the age of attainment of the CVM stage 3 [14]. On the contrary, herein the sagittal growth had also no influence on the age of attainment of the CVM stage 4, while it has been reported that this stage is reached by Class III subjects about 5 months later compared to Class I subjects [14]. Possible explanations for such contrasting evidence would reside in the multivariate analysis used herein or in the concept that in the present study only 23 subjects showed an ANB angle ≤0°; thus, a full comparison for Class III subjects has to be done with caution. Moreover, the entity of Class III malocclusion also has to be taken into account along the concept that previous investigations were limited to subjects with normal vertical growth, that is, normodivergent [14].

Interestingly, the only previous investigation [13] using multiple regression models on the age of attainment of different CVM stages and sagittal growth of the face reported no significant difference between the Class I and Class III subjects. However, this study [13] missed the reporting of data regarding vertical growth, and this parameter was used for adjustments in the multiple regression model. Therefore, the question whether in Class III malocclusion subjects the interval between the ages of attainment of the CVM stages 3 and 4 is longer than that in Class I subjects is still an open issue.

Even considering the duration of each CVM stage from 2 to 4 lasting 1 year, as initially proposed [1], inherent error in the use of such discrete staging systems would make reliable and clinically relevant a variation in age of the attainment of each circumpubertal CVM stage when of at least 4–6 months [16]. Considering the mean values of ANB, SN/MP, and NSBa angles seen herein and the corresponding *β* coefficients (even those close to the statistical significance), estimations of ranges for these craniofacial parameters, from which relevant age variation in the attainment of the CVM stages is expected, may be carried out. In particular, subjects with expected age variation of at least 6 months in the attainment of the different stages would be as follows: (i) for the CVM stage 2, those with an ANB angle at least ±3.8° of the sample mean of 3.7° (10.0% of the whole group); (ii) for the CVM stage 3, those with an SN/MP angle at least ±8.6° of the sample mean of 30.4° (11.3% of the whole group); and (iii) for the CVM stage 3, those with an NSBa angle at least ±10.0° of the sample mean of 130.0° (3.8% of the whole group). However, the actual duration of each CVM stage is subjected to variability in individual subjects [28] that may not be uncovered in cross-sectional investigations. While this variability would not compromise the results obtained by correlation analyses in a group of subjects, it has to be taken into account when dealing with individual patients, especially when little associations are seen. Moreover, unless raters undergo dedicated training [20], the repeatability of the CVM stage assignment may be not satisfactory [29]. A further limitation of the present study is related to the contrasting evidence regarding the reliability of the CVM method in detecting the mandibular growth peak [16, 28, 30–33]. However, most of the current studies used different variants of the CVM method [16, 33, 34], making results poorly comparable, or were focused on Class II malocclusion subjects [35], limiting the external validity. However, such conclusions may only be applied to the mandibular sagittal growth, with correlations of the CVM stage with vertical growth still poorly investigated. The present study warrants further investigations using different growth indicators, such as hand-and-wrist maturation [36] or third finger middle phalanx maturation [37] methods or longitudinal designs. Of note, while potential biases due to temporomandibular disorders were excluded herein, the present study was based on a population of subjects seeking orthodontic treatment; thus, the present results have to be extended with caution to general population without evident malocclusion.

## 5. Conclusions

Age variations in the attainment of the different circumpubertal CVM stages 2 to 5 have been seen mainly for vertical craniofacial growth pattern, as recorded through the SN/MP angle, with hyperdivergent and hypodivergent subjects, having an anticipated and delayed attainment of the pubertal CVM stage 3. However, such association would become clinically relevant only in extreme cases that would have a low prevalence in a population of subjects seeking orthodontic treatment of about 1 case out of 10. Timing for functional treatment of vertical discrepancy that requires to be performed during the pubertal growth spurt may take advantage of this evidence.

## Figures and Tables

**Table 1:** Chronological age for each CVM stage according to the sexes.

Sex	Cervical vertebral maturation stage group			
	CVM stage 2 (*N* = 80)	CVM stage 3 (*N* = 80)	CVM stage 4 (*N* = 80)	CVM stage 5 (*N* = 80)
Females	10.1 ± 1.2	11.5 ± 0.8	12.0 ± 1.2	12.7 ± 1.6
Males	11.4 ± 1.4	12.8 ± 1.5	13.3 ± 1.4	14.1 ± 1.2

*Diff.*	*0.000; S*	*0.000; S*	*0.000; S*	*0.000; S*

Each CVM stage group includes equal number of females and males. Data on age are presented as mean ± SD. Diff., significance of the difference between the sexes within each CVM stage group. S, statistically significant.

**Table 2:** Descriptive statistics for the craniofacial parameters (in degrees) for each group.

Parameter (degree)	Cervical vertebral maturation stage group				Diff.
	CVM stage 2 (*N* = 80)	CVM stage 3 (*N* = 80)	CVM stage 4 (*N* = 80)	CVM stage 5 (*N* = 80)	
SNA angle	80.7 ± 3.6	81.2 ± 3.5	80.3 ± 3.2	81.2 ± 3.4	0.301; NS
SNB angle	77.0 ± 3.9	77.2 ± 3.6	76.7 ± 3.7	77.7 ± 3.7	0.365; NS
ANB angle	3.7 ± 2.0	3.9 ± 2.1	3.6 ± 2.2	3.5 ± 2.3	0.558; NS
SN/PP angle	7.0 ± 3.4	7.1 ± 3.9	7.9 ± 3.3	8.1 ± 2.8	0.200; NS
SN/MP angle	31.2 ± 5.6	30.4 ± 5.9	30.8 ± 5.9	30.6 ± 5.3	0.848; NS
NSBa angle	129.6 ± 5.2	129.4 ± 4.6	130.0 ± 4.9	130.7 ± 4.7	0.375; NS

Each CVM stage group includes equal number of females and males. Data on age are presented as mean ± SD. Diff., significance of the levels of differences among the CVM stage groups for each cephalometric parameter. NS, not statistically significant.

**Table 3:** Results of the backward multiple linear regressions for the association of craniofacial cephalometric parameters with the chronological age (in months) for each CVM stage.

Explanatory variable	*β* (SE)	*t*	Sig.
*Model 1: age of attainment of CVM stage 2 (N = 80), R* ^*2*^ * = 0.213*			
Sex (male)	17.0 (3.6)	4.754	0.000; S
ANB angle	1.6 (0.9)	1.793	0.077; NS
*Model 2: age of attainment of CVM stage 3 (N = 80), R* ^*2*^ * = 0.269*			
Sex (male)	13.6 (3.2)	4.311	0.000; S
MP/SN angle	−0.7 (0.3)	2.477	0.015; S
*Model 3: age of attainment of CVM stage 4 (N = 80), R* ^*2*^ * = 0.194*			
Sex (male)	15.4 (3.6)	4.339	0.000; S
NSBa angle	0.6 (0.4)	1.700	0.093; NS
*Model 4: age of attainment of CVM stage 5 (N = 80), R* ^*2*^ * = 0.165*			
Sex (male)	16.3 (4.0)	4.080	0.000; S

Independent variables entered in each model: sex, SNA angle, ANB angle,  SN/MP angle, and NSBa angle, with variables having a *p* value above 0.1 removed from the model. Results of the multiple linear regressions are presented as *β* (SE); *R* ^2^, coefficient of determination. Sig., level of significance; S, statistically significant; NS, not statistically significant.
